# The Medical and Surgical Register: Consisting Chiefly of Cases in the New-York Hospital

**Published:** 1821-02

**Authors:** 


### CRITICAL ANALYSES

OF

### RECENT PUBLICATIONS, IN THE DIFFERENT BRANCHES OF MEDICINE AND SURGERY,

### In the Literature of Foreign Nations.

---

Πατρίδες ἄρα
Ανδράσιν, ἃ πατραῖς ἄνδρες, ἀγαλλόμεθα.

---

*The Medical and Surgical Register: consisting chiefly of Cases in the New-York Hospital.* By John Watts, jun. m.d. Valentine Mott, m.d. and Alexander H. Stevens, m.d. Part II. Vol. I. 8vo. pp. 240. Collins and Co. New-York; and Souter, London. 1820.

THE case first in order is that of "*An extraordinary Tumor, successfully Extirpated,*" by Dr. Stevens.

The principal remark we have to make respecting this case, is that it presents a very favourable specimen of the degree of perfection to which operative surgery has been raised in the United States. The tumor in this case seems to have been of a carcinomatous kind: for "a scalpel pushed into it, imparted to the fingers the grating feel of brisket, or intervertebral substance;" and "it contained several cavities, from which a colourless fluid was discharged." It extended laterally from the situation of the mastoid process on the right side, beneath the tongue and the cavity of the mouth, to one inch beyond the symphisis of the lower jaw, two inches beyond the trachea on the left side, and downwards to two inches and a half below the clavicle. It hung pendulous externally to the integuments of the neck, excepting at its base, which was attached, as already indicated, to the parts bounded by the mastoid process, the floor of the mouth, the larynx and two inches beyond it on the opposite side, and the inner edge of the sterno-cleido mastoid muscle. Its original adhesion to the parts in this course had probably been increased by the inflammation produced by an awkward and unsuccessful attempt at its extirpation on a former occasion by another surgeon. It weighed, after the fluid it contained was evacuated, three pounds and a half. The dexterity and professional ability manifested in the operation, will be shown by stating that the wound left by the operation "exhibited the pulsation of the external carotid; the ninth pair of nerves; the digastric, the stylo-hyoid, and the mylo-hyoid, muscles; and in general all the external layer of muscles which elevate or depress the os hyoides and the cartilages of the larynx;" and that the operation was effected without the loss of any considerable quantity of blood, (but which was nevertheless sufficient to make Dr. Stevens advert to the "advantage of cutting and securing the trunks of vessels, before their branches are divided," on similar occasions,) and with the most favourable result.

The next case is one of "*Extensive Infiltration of Urine successfully treated,*" by Dr. STEVENS. This was an ordinary case of infiltration of the scrotum and adjacent parts, from rupture of the urethra consequent on stricture. The most interesting remarkable circumstance respecting it, is the recovery of the patient, although five days had elapsed since the rupture occurred before he was brought into the hospital, and before any scarifications were made in the integuments; so that extensive mortification had already commenced when this measure was first resorted to.

Dr. Stevens remarks on this case, that it illustrates "the mischievous effects of violence in the use of the catheter, as tending to produce a rupture in the urethra;" but it does not appear, from the history of the case, that the use of the catheter had any thing to do with the production of the "mischievous effects," or that a catheter had ever been used previously to the occurrence of the bursting of the urethra, which seems to have happened in the ordinary way. The patient had a stricture, which became gradually worse, and one day, on "straining to empty his bladder, he felt that the bladder was partially emptied; for, although no water was discharged from the meatus urinarius, his scrotum and perineum began to swell."

Dr. Stevens then takes occasion to reprobate "the looseness with which writers describe the degree of force which should be employed, in the various operations of surgery, in directing *moderate* force to be employed;" and he suggests, by way of amendment, that "it were better if surgeons should say to their pupils, you may employ for the reduction of a hernia, a force equal to the weight of ten pounds; you may employ for the extension in the reduction of a dislocated arm, a force equal to the weight of fifty pounds; you may pull upon the cord with a force equal to the weight of three pounds; you may push in the catheter with a force equal to the weight of one pound." If we were disposed to wonder at any thing, we should wonder that a *practical* surgeon could suggest such precepts. They are like the speculations of a man not only unversed in clinical experience, but also unacquainted with the physiology of the human body. A disciple of Borelli and the mechanical physiologists would hardly have fallen on such a project. Were we, also, disposed to urge particular objections to the precepts which Dr. Stevens has adduced as examples of his proposed means of instruction, we should argue for the inadequacy, in the generality of instances, of the force he has proposed as proper for attaining the object in view, in at least one of the cases: thus, a degree of force equal to the weight of fifty pounds would be useless in almost every instance, in an adult, as a means of extension for the reduction of a dislocated arm; and, instead of one pound being the extent of force allowable in the introduction of the catheter, we believe that there are many surgeons, who are considered good ones too, who would think force to an extent equivalent to twenty pounds too confined a restriction in cases of very frequent occurrence.

"*Remarks on the Treatment of Secondary Syphilis;*" by A. H. STEVENS, M.D. The principal object of this paper is said by the author to be "to make known the results of the writer's experience in the New-York Hospital, in the use of a compound syrup of sarsaparilla and guiacum, for the cure of what are termed cases of secondary syphilis, and other severe forms of disease arising from the action of mercury, in constitutions predisposed to scrofula." There is nothing in the author's observations that is either new or calculated to be particularly interesting to the English practitioner. We shall, however, extract the following passage, as it presents a summary of the results alluded to.

"The difficulty of distinguishing cases that require a further use of mercury, from those in which disease is kept up by the continued action of that medicine, after the administration of it has ceased, has led me to adopt as a rule, never to prescribe it where it has been previously given in unascertained quantities; and three-fourths of the cases I have met with have been immediately relieved, and, to the best of my knowledge, ultimately cured, by the use of the preparation alluded to and the decoction of sarsaparilla, without the necessity of a further use of mercury."

"*A Case of Contraction of the Vagina, from Sloughing caused by a tedious Labour, in which the Cicatrix was safely divided by a Bistoury to facilitate Parturition during a subsequent Labour;*" by Dr. STEVENS. The title of this paper sufficiently indicates its chief practical interest. The case comes under the class of those which the sagacious physician of the Pyrenees described to Bordeu as "un tissu de menus détails, de petits faits, dans le cas d'être prévus par les connoisseurs ou du moins bien traités lorsqu'ils se présentent."

"*A Case of Arm Presentation, attended with untoward Circumstances, in which the Child was extracted in a peculiar manner;*" by Dr. STEVENS. The extraction was accomplished by employing force so as to make the body of the child turn in a manner similar to what it does in what is called the "spontaneous evolution," when the breech is expelled before the head in an arm presentation. This was effected by "making an incision (the child being dead) as high up on the back as I safely could," says Dr. Stevens, "with a blunt bistoury passed along the fore-finger; inserting a blunt hook into it, and pulling in a direction obliquely downwards and forwards, (the back of the child presenting to the abdomen of the mother,) so as to bring the buttocks down; and, having perfectly succeeded, I made a second incision, immediately above the hip, introduced the hook as before, and extracted the child, breech foremost."

"*Remarks on the Means of arresting the Course of the Blood through the Tibial Arteries;*" by Dr. STEVENS. The principal object of this article is to recommend the use of a form of the tourniquet invented by Dr. MOORE, of Massachusetts, with the addition of a splint suggested by the author himself, for the purpose of arresting the circulation in the tibial arteries. We have not yet received the Journal which contains the description of Dr. Moore's tourniquet, and must, therefore, defer until a future time any remarks we have to offer in this paper.

"*Remarks on Fistula Lachrymalis;*" by Dr. STEVENS. This article contains merely an account of Mr. Dupuytren's mode of operating for the cure of the disease above named, which has already been described in a late Number of this Journal.

"*A Case of Crural Hernia;*" by Dr. STEVENS. The history of this case presents nothing new; but Dr. Stevens takes the opportunity which the narration of it offers, for making some remarks on the risk "of cutting the intestine by the incision upwards, as recommended by Gimbernat, Lawrence, and others," and of recommending the use of an instrument of his invention, for the division of the crural ligament, which Dr. Stevens does not appear to have hitherto employed. It has some analogy with the gorget, both in its figure and in the mode in which it effects the requisite incision; and, in saying this, we believe we state what is sufficient to show that it never will be employed by any good surgeon, if the inventor of it be, perhaps, excepted.

"*Cases of Gonorrhœa, cured by Cubebs;*" by Dr. STEVENS. These cases, from the length of their duration, (fifteen days being the shortest period,) under the use of the cubebs, appear to us to present evidence disadvantageous to the reputation of the remedy: the results were certainly much less favourable to it, excepting that no positive mischief seems here to have occurred from its administration, than those which have been published in this country.

The whole of the book before us is not occupied by articles of such little value as those which have hitherto been noticed. The paper next in order is by Dr. WATTS, and it, like the other writings of this physician, contains matters of interest and importance, selected and arranged by a well-cultivated taste, and reasoned on, for the most part, with sound judgment. This paper occupies above 150 pages, and presents an excellent account of the yellow-fever, as it appeared in various parts of the United States in the year 1819; with some reflections, of a general character, on the history, nature, and treatment, of that disease, and on the policy of the American government respecting the means of preventing it. As an abstract of this dissertation was given in the *Proëmium* to the present volume of this Journal, in conjunction with similar accounts of several other treatises on the same subject, we shall not here enter into the review of it; though we are disposed to remark, that Dr. Watts appears to us to have, in two instances, drawn inferences which are not sufficiently well supported by facts. The first of which is, that the yellow-fever is "an aggravated form of bilious remittent fever;" the second, that it is *never* propagated by contagion. It is shown in the compilation above referred to, that evidence has been wanting, in many cases, of the existence of either organic lesion or particular functional disorder in the liver; and, though the endemic origin of the disease in the United States seems to have been proved in the most satisfactory manner, there is still probable evidence that it has, in some instances, been communicated by means of contagion, after having originated in the way just designated. The notion of the casual origin of animal poison in the way here indicated, is, we are aware, regarded as an unphilosophical inference by some men of eminent talents; but it is, nevertheless, a notion which is entertained by almost every modern physician who has had much experience in the observation of the *fevers* of Europe, to which the notion is, by them, applied; and, if it is true with respect to the typhous fever of Europe, there are good grounds for inferring, by analogy, that it is also true with respect to the yellow-fever of America and the Antilles.

"*Further Remarks on the Case of Ligature of the Arteria Innominata;*" by VALENTINE MOTT, M.D. Although Dr. Mott makes no particular allusions, it seems that these remarks are adduced in consequence of its having been argued that the case in question was not one of aneurism. As we ourselves expressed such an opinion, (see the Number of this Journal for August, 1819,) we shall transcribe here the whole of those remarks.

"In my first communication of this case, in the Hospital Register for 1818, it is stated (page 50) that 'the subclavian artery, internally and externally to the disease, was pervious.' To this it may now be added, that, where this artery opens into the ulcer left from the wound of the operation, it appears not only pervious, but of the natural size, and the coats free from any diseased appearance. Externally towards the axilla, the artery is somewhat enlarged in diameter, but exhibits no appearance of disorganization of its coats, either externally or internally. About an inch from the ulcer, or just as the artery has passed between the scaleni muscles, there is an irregularly-shaped elliptical opening upon its upper side, large enough to receive the extremity of the fore finger. The edges of this opening are jagged and uneven, and the surface of the artery internally is of a brownish-yellow colour, to the extent of half an inch on the inside of the opening, and more than an inch towards the axilla. The internal coat of the artery has a rugous or puckered appearance; separated a little from the muscular coat, very friable, and evidently in a degenerated state. The opening of the artery communicates directly with the anterior extremity of the sac, which contains coagula; and, upon removing these, the surface of the sac is seen puckered, or thrown into a great number of little folds, giving it at first sight the appearance of containing a number of holes.

"This account is taken from the morbid parts before me; and the preparation has been seen and examined by Dr. Post, Dr. Hosack, Dr. Stevens, Dr. Watts, and others, who have authorized me to state that they are satisfied as to the nature of the case."

"*Two Cases of disunited Fractures, successfully treated by Seton;*" by Dr. MOTT. The results of former experience in the practice here designated, were stated in a late Number of this Journal, (Nov. 1818.) We shall transcribe the accounts of these cases in detail.

"CASE I.—Stephen Hammond, aged about 35 years, was admitted into the hospital with lameness, in consequence of a fracture of the leg about seven months since. Upon examination, the tibia was found un-united, and to admit of very free motion between its ends. The fibula was entire, and the patient believed it never had been broken. From the account which he gave, it appeared that he had been subjected to the proper treatment for the restoration of a broken bone; but he stated that it never showed any disposition to unite, under the course which was pursued.

"As his general health was not good, he was put upon tonic medicines and invigorating diet; and I directed that a stimulating plaster of gum-ammoniac and mercury should be applied over the part, with the many-tailed bandage; and splints to reach above the knee and below the ankle, and to be very firmly secured; and I advised him to walk upon it with the assistance of his crutches, as much as the pain would any way permit; informing him that the object in wishing this exercise was to inflame and irritate the ends of the bone, and that he must not desist, even though considerable pain should accompany it. This was persevered in for several weeks; but, finding little or no pain to attend it, and no appearance of inflammation in or about the fracture, and no hope of amendment, it was discontinued. Blisters were next repeatedly tried, but to no purpose. Very powerful shocks of electricity, also, were passed in different directions through the part, but they produced no beneficial effects.

"A seton was next introduced. This I did by making a small incision upon the outside of the tibia, down to the fractured ends; then passing between the bones the stilette of a small trocar, and pushing it out on the opposite side, the seton was readily introduced with an eyed probe.

"In a few days considerable inflammation and pain supervened, which required emollient poultices and the antiphlogistic treatment to subdue. This was soon followed by a copious discharge of matter from the seton, and a collection of pus on the anterior part of the tibia, which was evacuated by a small incision. After five or six weeks, he became sensible of an increase of firmness in the leg; and from this time he was directed to diminish the size of the seton one thread every other day, until it was all removed. It continued to grow stronger every day; and, in a short time after the wounds healed, he was permitted to walk a little upon it, when splinted and tightly bandaged; and in about three months the bone was firmly united.

"CASE II.—John Smith, aged 41 years, became a patient in the hospital in 1819, in consequence of an un-united fracture of the thigh-bone, of twelve months standing. It occurred at sea, and at the same time several of his ribs were fractured. Thirty-six days after the accident he arrived at Halifax, without having had any attention paid to the adjustment of the bones. After his arrival, he states that little notice was taken of his thigh, and no attempt was made to reduce it. He recovered without difficulty from the fracture of his ribs. The several means mentioned in Case I. were tried, but without benefit. The Limb was considerably shortened, from the obliquity of the fracture and ends of the bone overlapping. No advantage attending the use of the means referred to; a seton was recommended. In the introduction of this, much more difficulty was experienced than in the case of Hammond.

"An incision was made on the inside of the thigh, a little to the outerside of the artery, so as to come down upon the centre of the ends of the bones, where they overlapped. The stillette was then attempted to be passed between the bones; but this was found altogether impracticable, from their very close contact, even though the limb was changed from one position to another. Instruments of different sizes were resorted to, but they could only be made to pass a very small distance. A gimblet was tried, but very little progress could be made. Having provided for the occasion a *carpenter's bit*, about the size of a large trocar, I found with this a passage could be made with the greatest facility. Then, by making an incision down to the end of the instrument, on the outside of the thigh, a large seton was readily conveyed through between the bones, by means of a long eyed probe.

"After the expiration of three months, the thigh becoming firmer, and much less motion being felt between the ends of the fracture, he was permitted gradually to lessen the size of the seton. The firmness continued regularly to increase; but it was not until after eight months had elapsed, that the thigh had acquired sufficient firmness to enable him to support the weight of his body, by the aid of a crutch.

"It is now more than twelve months since the seton was introduced, and the bone appears to be firmly united. The shortening of the limb does not exceed three inches and a half."

"*A Case in which the Right Carotid Artery was tied, for the safe Removal of a Tumor;*" by Dr. Mott. The practice alluded to in the title of this paper, seems to be favourably regarded by the generality of modern surgeons. The few points of an interesting character in the history of this case could not be stated, without entering into details which our limits will not admit of. We should however remark, that the operation, which was one of much difficulty, appears to have been performed with great dexterity; but, when we consider the able and successful manner in which a ligature was applied to the arteria innominata by Dr. Mott,—an operation which, considering its extreme difficulty, as well as its object, we regard as one of the most admirable specimens of operative surgery,—we can expect nothing from him but manifestations of great abilities in this department of the healing art.

The last article in this volume is an account of "*a Case of Carcinoma of the Penis cured by Amputation, and subsequent Removal of the Inguinal Glands, successfully;*" by Dr. Mott. The remarks we made respecting the last case are equally well applicable to this also.

